# Protective and remineralizing capacity of biosilicate, chitosan-based material, and fluoride on demineralized and irradiated enamel

**DOI:** 10.1007/s00411-026-01234-7

**Published:** 2026-06-23

**Authors:** Kelly Fernanda Molena, Vicente Silva Mattos, Jarbas Caiado de Castro Neto, Francisco Wanderley Garcia de Paula-Silva, Harley Francisco de Oliveira, Regina Guenka Palma-Dibb, Fernanda de Carvalho Panzeri, Alexandra Mussolino de Queiroz

**Affiliations:** 1 Department of Pediatric Dentistry, School of Dentistry of Ribeirão Preto, University of São Paulo, Ribeirão Preto, São Paulo, Brazil; 2 São Carlos Institute of Physics, University of São Paulo, São Carlos, Brazil; 3 Department of Restorative Dentistry, School of Dentistry of Ribeirão Preto, University of São Paulo, Ribeirão Preto, São Paulo, Brazil; 4 Department of Dental Materials and Prosthodontics, School of Dentistry of Ribeirão Preto, University of São Paulo, Av. do Café - Subsetor Oeste - 11 (N-11), Ribeirão Preto, São Paulo, 14040-904 Brazil

**Keywords:** Dental Caries Susceptibility, Radiotherapy, Tooth Remineralization, Biosilicate, Chitosan, Acidulated Phosphate Fluoride

## Abstract

This study aimed to investigate the protective and remineralizing capabilities of biosilicate, chitosan, and fluoride on demineralized enamel, pre- and post-radiotherapy (RT). Enamel samples (*n* = 180) underwent artificial caries process (ACP) and 70 Gy irradiation. They were divided into ten groups (*n* = 18): Biosilicate Suspension Pre-RT (G1), Post-RT (G2); Biosilicate Gel Pre-RT (G3), Post-RT (G4); Chitosan Gel Pre-RT (G5), Post-RT (G6); Fluoridated Gel Pre-RT (G7), Post-RT (G8); Caries Control + RT (G9); No Caries Control + RT (G10). Knoop microhardness and surface roughness were measured pre- and post-ACP and post-treatment. Scanning Electron Microscopy (SEM) and Atomic Force Microscopy (AFM) evaluated topography. Energy Dispersive X-ray Spectroscopy (EDS) and Raman Spectroscopy assessed chemical composition. ACP caused enamel prism disruption. G10 had the highest microhardness and lowest roughness (*p* < 0.05). G3 and G5 performed better pre-RT; G4 and G8 performed better post-RT. SEM and AFM showed surface protection and particle deposition in the treated groups. No significant chemical differences were found in EDS and Raman analyses. Biosilicate and partially preserved enamel microhardness and offered protective effects, alongside fluoride. These materials may benefit head and neck cancer patients by forming a protective layer and preventing radiation-related enamel demineralization.

## Introduction

Head and neck cancer (HNC) comprises approximately 10% of all malignant tumors in developed countries and represents a major global health concern (Johnson et al. 2020). Patients with HNC frequently present compromised oral health, which may complicate dental management before oncological treatment (Critchlow et al. 2014; Patel et al. 2020). Therapeutic approaches typically include surgery, chemotherapy, and radiotherapy (RT) (Horton et al. 2019; van Harten, and Brakenhoff 2021. Although RT is effective in achieving local tumor control, it is commonly associated with adverse oral effects such as mucositis, xerostomia, opportunistic infections, trismus, and osteoradionecrosis (Ferlay et al. 2012; Brook 2020). Furthermore, RT induces alterations in mineralized dental tissues, leading to enamel demineralization and structural degradation, including hydroxyapatite crystalline disorganization, increased interprismatic porosity, and greater susceptibility to acid demineralization, potentially associated with radiolysis-related physicochemical alterations induced by ionizing radiation (Gonçalves et al. 2014; Santin et al. 2015; Queiroz et al. 2019; Kudkuli et al. 2020; Arid et al. 2020, 2024; Conti et al. 2022; Barbosa et al. 2023; Dos Santos et al., 2024), thereby predisposing patients to radiation-related caries (RRC) (Jongebloed et al. 1988; Silva et al. 2010).

RRC differs from conventional caries in both localization and progression, predominantly involving cervical, cusp, and incisal surfaces (Jongebloed et al. 1988). The progression of enamel loss to dentin exposure (Queiroz et al. 2019), coupled with the occurrence of recurrent lesions, compromises treatment outcomes and negatively impacts quality of life (Lee et al. 2021; Yuwanati et al. 2021). Recent physicochemical evidence also suggests that radiation may directly alter the structural and chemical properties of dental tissues, supporting the existence of non-salivary mechanisms contributing to radiation-related caries development (Tamahara and Kouketsu 2025). Consequently, implementation of preventive dental care before RT, together with long-term clinical follow-up, is essential (Silva et al. 2010; Gupta et al. 2015; Lee et al. 2021; Dos Santos et al., 2024). Additionally, daily home care strategies, including fluoride use, xerostomia management, and remineralizing agents, are strongly recommended (Thariat et al. 2012; Gupta et al. 2015).

Topical fluoride is widely used against RRC (Horiot et al. 1983; Thariat et al. 2012; Hong et al. 2018), but suffers from poor adherence (Thariat et al. 2012), especially due to xerostomia and discomfort. Moreover, fluoride may cause surface degradation of restorations (McComb et al. 2002; De Moor et al. 2011) and fail to fully protect irradiated enamel (Lopes et al. 2018). Previous studies suggest that fluoride primarily acts at the enamel surface through fluorapatite formation and may present limited effectiveness against deeper radiation-induced structural alterations in enamel (Wu et al. 2020; Yao et al. 2024). Therefore, alternative and complementary strategies are needed, particularly strategies capable of preserving enamel microhardness, maintaining surface integrity, and preventing structural degradation after irradiation (Gonçalves et al. 2014; Queiroz et al. 2019; Brook 2020).

Biosilicate, a bioactive glass-ceramic, promotes ionic supersaturation and hydroxyapatite nucleation through calcium and phosphate ion release, favoring apatite crystal growth and mineral deposition on the enamel surface, thereby promoting remineralization (Tirapelli et al. 2011; Chinelatti et al. 2017). Chitosan, a biocompatible polymer with bioadhesive properties, may favor mineral retention and surface stabilization through electrostatic interactions with enamel (Nimbeni et al. 2021; Yamakami et al. 2022; Rafiee et al. 2024), and stimulates salivation in vivo (Sionov et al. 2021). When combined with casein phosphopeptides (CPP), it may enhance remineralization by stabilizing phosphate and calcium (Yamakami et al. 2022). Although effective in reducing mineral loss, this combination alone does not restore surface hardness (Sionov et al. 2021). Nanohydroxyapatite promotes enamel repair by depositing apatite nanoparticles and maintaining mineral supersaturation (Pushpalatha et al. 2023). Thus, the combined use of chitosan, casein, and nanohydroxyapatite has not been studied in irradiated enamel.

Considering the bioactive behavior of these materials and their physicochemical interactions with enamel, biosilicate was expected to promote ionic supersaturation and hydroxyapatite precipitation through calcium and phosphate ion release (Panzeri Pires-de-Souza et al. 2015; Chinelatti et al. 2017; Abreu et al. 2023), while chitosan-based formulations were expected to favor surface stabilization and mineral retention through electrostatic interactions and biomimetic mineral deposition (Nimbeni et al. 2021; Yamakami et al. 2022; Lopes et al. 2023). Fluoride, in turn, was expected to increase enamel resistance to acid dissolution mainly through superficial fluorapatite formation (Wu et al. 2020; Yao et al. 2024).

From a broader global health perspective, improving preventive strategies for patients undergoing cancer therapy is aligned with the United Nations Sustainable Development Goals (SDGs), particularly SDG 3, which aims to reduce premature mortality from noncommunicable diseases and improve long-term health outcomes worldwide. Cancer is a major contributor to the global burden of noncommunicable diseases, and strengthening prevention and supportive care strategies is considered essential for achieving SDG target 3.4 (Morgan et al. 2018; Niessen et al. 2018). In this context, the development of preventive approaches capable of minimizing radiotherapy-related oral complications may contribute to improving quality of life and long-term health outcomes in oncology patients. Therefore, it was hypothesized that bioactive remineralizing agents with distinct physicochemical mechanisms would partially preserve enamel structure and mechanical properties after artificial caries challenge and radiotherapy, with different patterns of surface interaction and mineral deposition among the evaluated materials. Considering the emergence of new materials that promote dental remineralization and inhibit the development of dental caries, this study aimed to evaluate the protective and remineralizing effects of Biosilicate, a chitosan-based formulation, and fluoride on enamel subjected to radiotherapy by analyzing microhardness, surface roughness, and surface morphology.

## Materials and methods

### Sample selection

A pilot study was conducted using 20 specimens (two per experimental group) to estimate variance parameters for enamel microhardness and surface roughness. Based on these preliminary data, sample size calculation was performed using OpenEpi software (Version 3.01, Open-Source Epidemiologic Statistics for Public Health). The calculation was based on comparison of mean values among experimental groups available in OpenEpi software, considering a 95% confidence interval (α = 0.05) and 80% statistical power. The minimum required sample size was determined to be 18 specimens per group. Accordingly, 18 specimens were included in each experimental group in the final analysis.

The bovine incisors were obtained from a commercial slaughterhouse supplier through institutional acquisition by the School of Dentistry of Ribeirão Preto, University of São Paulo (FORP/USP). The teeth originated from animals processed for the food industry, and therefore no animals were sacrificed specifically for research purposes, which dispensed the need for approval by an animal ethics committee according to institutional regulations. The selection criteria requested to the supplier included incisors with complete root formation, preserved crowns, and absence of cracks, fractures, discoloration, or severe wear. All teeth were cleaned with periodontal curettes and immersed in 1% sodium hypochlorite for 24 h, for disinfection purposes and removal of residual organic debris prior to specimen preparation. After disinfection, the teeth were thoroughly rinsed and stored in 80 mL universal containers containing saline solution until experimentation. Teeth were then sectioned into standardized enamel fragments (6 × 6 × 2 mm) from the central crown area using a metallographic cutter (Isomet 1000, Buehler, Lake Bluff, IL, USA), and polished sequentially with silicon carbide papers (#400, #600, #1200; Norton, Guarulhos, SP, Brazil) under irrigation. Enamel surface roughness was standardized to ≤ 0.2 μm and measured with a rugosimeter (Surfcorder SE 1700, Kosakalab, Tokyo, Japan). Three readings (2.4 mm length, 0.8 mm cut-off, 0.25 mm/s speed) were obtained per specimen: one at the center and two positioned 1 mm from the midpoint on opposite sides.

#### Treatments

The samples were randomly separated into 10 groups (*n* = 18) according to the treatment submitted: G1: Biosilicate Suspension Pre-RT; G2: Biosilicate Suspension Post-RT; G3: Biosilicate Gel Pre-RT; G4: Biosilicate Gel Post-RT; G5: Chitosan Gel Pre-RT; G6: Chitosan Gel Post-RT; G7: Fluoridated Gel Pre-RT; G8: Fluoridated Gel Post-RT; G9: Caries Control + RT; G10: No Caries Control + RT. Groups G9 and G10 did not receive any protective or remineralizing treatments.

#### Biosilicate suspension

The biosilicate suspension was prepared by mixing 0.15 mg of biosilicate powder (Vitrovita, São Carlos, SP, Brazil) with 1.35 mL of distilled water, followed by stirring for 1 min. The solution was applied to the enamel surface using a microbrush and left undisturbed for 5 min (Abreu et al. 2023). Subsequently, the enamel surface was rinsed with water for 30 s, and the excess was gently removed using gauze.

#### Biosilicate gel

The biosilicate gel consisted of 1.8 mg of biosilicate incorporated into 18 mL of carboxymethylcellulose gel at pH 7. After 1 min of mixing, the gel was applied to the enamel using a microbrush and spread for 1 min. It was allowed to act for 5 min (Abreu et al. 2023), followed by rinsing with water for 30 s and removal of any residue with gauze.

#### Chitosan gel

The chitosan-based gel was composed of 1.5% chitosan (75–85% deacetylated, Sigma-Aldrich, Barueri, Brazil), bovine casein gel (C3400, Sigma-Aldrich), and modified nanohydroxyapatite (Sigma-Aldrich). The gel was actively applied to the enamel with a microbrush for 10 s and left in contact with the surface for 4 min (Yamakami et al. 2022). Excess was removed using gauze.

#### Fluoride gel

The acidulated phosphate fluoride (APF) gel used contained 1.23% fluoride (DFL Indústria e Cosméticos, Brazil; lot number 22121248; expiration date December 2024). The gel was actively applied with a microbrush for 1 min, allowed to remain on the enamel for 2 min, and then the excess was removed with gauze, accordingly to manufacturer’s recommendations.

### Artificial Caries Process (ACP)

All fragments, except those in G10, were subjected to an Artificial Caries Process (ACP), consisting of an in vitro demineralization protocol designed to simulate subsurface enamel caries lesions under controlled laboratory conditions, according to Moron et al. (2013). All surfaces of the samples were coated with cosmetic nail polish, leaving only the enamel surface exposed to the demineralization process. The specimens were fixed to a container using nail polish and covered with a 0.5 cm-thick layer (equivalent to 1.5 mL) of 6% carboxymethyl cellulose gel (pH 5.0). The gel remained in contact with the enamel surface for 12 h at 4 °C.

Subsequently, 1.5 mL of 0.1 M lactic acid (pH 5.0) was added to the samples and maintained for 14 days at 37 °C. At the end of the demineralization cycle, the specimens were rinsed with distilled water, dried with absorbent paper, and then stored in artificial saliva at 37 °C. The artificial saliva used in this study consisted of a manipulated synthetic saliva base prepared by Farmácia da Terra (Ribeirão Preto, SP, Brazil), containing sodium benzoate, calcium chloride, magnesium chloride, potassium chloride, sodium chloride, sodium fluoride, preservative agents, sorbitol, phosphate compounds, and purified water.

#### Irradiation protocol

All specimens were subjected to conventional radiotherapy using Intensity-Modulated Radiation Therapy (IMRT), following a standard fractionation protocol with a total dose of 70 Gy delivered in 35 fractions (2 Gy/day, five times per week, over seven weeks). The total dose of 70 Gy was selected because it corresponds to a conventional cumulative therapeutic dose commonly used for head and neck cancer radiotherapy, particularly for tumors requiring definitive or adjuvant treatment. Therefore, this protocol was intended to reproduce clinically relevant enamel exposure conditions for teeth located within or near the irradiated field.

During irradiation, samples were individually placed in acrylic cell culture plates (Cellstar, ref. 657160, Greiner Bio-one, Frickenhausen, Germany) to ensure uniform dose distribution across their surface. To simulate the moist oral environment, each well was filled with 10 mL of distilled water, which was replaced after each session; between irradiations, specimens were stored in artificial saliva at 37 °C. The use of distilled water during irradiation minimized interference with dose distribution due to the high viscosity and ionic concentration of artificial saliva, while still reproducing the physical and chemical radiotherapy effects associated with free radical generation. This approach was adopted to minimize ionic exchange during irradiation, following protocols previously described in studies evaluating radiation-induced enamel alterations (Santin et al. 2015; Dos Santos et al. 2024).

Irradiation was performed using a 6 MV photon linear accelerator (Elekta Synergy Platform; Elekta, Stockholm, Sweden), with four acrylic plates irradiated simultaneously in a 20 × 40 cm radiation field. Samples were positioned on a carbon fiber table, equidistant from the beam center, to ensure a uniform dose rate of 600 cGy/min. The fractional dose of 2 Gy was calculated and administered at the mid-depth of the samples, using a single-field setup based on plate thickness. Monitor units (MUs) were determined by a certified medical physicist, accounting for calibration, scattering, and field size. Quality assurance followed the IAEA TRS 398 dosimetry protocol and Brazilian National Nuclear Energy Commission regulations (CNEN Norma CNEN NN 6.10; Resolution CNEN 277/21). The irradiation protocol reproduced clinical conditions applied to head and neck cancer patients, and all procedures were monitored by a trained technician.

#### Microhardness

Knoop Microhardness readings were conducted at three time points: L1 (pre-ACP), L2 (post-ACP), and L3 (post-RT and treatments), with a Microhardness Tester HMV-2 (Shimadzu, Tokyo, Japan), under a static vertical load of 25 g applied for 5 s. All measurements were performed by a single previously trained examiner following pilot standardization under technical supervision in order to minimize inter-examiner variability and improve measurement reproducibility.

For each specimen, three initial readings were performed at different locations, following the same pattern used for surface roughness measurements. The mean value was considered as the baseline (KHNi) Data were calculated by the formula (Amorim et al. 2024):

$$\:KHN=\frac{1.451}{{d}^{2}}F$$

Where (KHN) is the Knoop hardness value, (F) is the test load (25 gf), and (d) is the length of the longest diagonal in the indentation. After treatments, final microhardness readings were obtained (KHNf) and the microhardness alteration (KHN%) was calculated using the formula:

$$\:\mathrm{K}\mathrm{H}\mathrm{N}\mathrm{\%}=\:\frac{({KHN}_{f}-{KHN}_{i})}{{KHN}_{i}}x100$$

Where KHNf is the final microhardness mean value and KHNI is the initial one.

The remineralization potential of the treatments was calculated by the formula (Amorim et al. 2024):

$$\:Potential\left(\%\right)=\frac{\:(KHNf-KHN\mathrm{c})}{(KHNi-KHN\mathrm{c})}$$

Where KHNc is the microhardness mean value after the ACP.

### Roughness surface

After treatments, final surface roughness was performed as previously described, at three time points: L1 (pre-ACP), L2 (post-ACP), and L3 (post-RT and treatments). Surface roughness alteration (ΔRa) was calculated using the formula (Amorim et al. 2024):

$$\:{\mathrm{D}R}_{a}=\:{R}_{af}-{R}_{ai}$$

Where Raf represents the final surface roughness and Rai the initial one.

#### Scanning Electron Microscopy (SEM)

A total of 40 samples (four per group) were mounted on aluminum stubs using double-sided carbon tape (Electron Microscopy Sciences, Washington, PA, USA) and coated with gold in a sputter coater (SDC 050; Bal-Tec, Balzers, Germany). Analyses were performed with a Jeol JSM 6610 LV scanning electron microscope (Tokyo, Japan) at magnifications of 500×, 1000×, and 2000×, covering the entire enamel thickness for morphological and qualitative comparisons (Dos Santos et al. 2024).

#### Atomic Force Microscopy (AFM)

A total of 20 samples (two per group) were analyzed using an atomic force microscope (Multimode 8-HR, Bruker, USA) operating in PeakForce Tapping mode to characterize the nanoscale topography of the vestibular surface (middle third) of the teeth. The instrument specifications included a 10 μm × 10 μm XY drive, a 4 μm Z drive, a 16-bit Nanoscope V controller, a manual sample displacement stage (2 mm × 2 mm) in XY, an environmental chamber for relative humidity control (5% to ambient), and a 10× objective lens. Prior to imaging, teeth were cleaned with pumice paste, and scans were performed over 2 μm × 2 μm areas at a resolution of 512 × 512 pixels and a scan rate of one line per second, using ScanAsyst-Air probes (Bruker, USA) with a nominal force constant of 0.4 N/m and a tip radius of < 10 nm. Data were processed with Gwyddion software (version 2.65), enabling the generation of two- and three-dimensional images saved in TIF format.

### Chemical analysis

#### Energy Dispersive X-ray Spectroscopy (EDS)

Qualitative and quantitative chemical analyses were conducted to identify elemental composition in enamel using energy-dispersive X-ray spectroscopy (EDS) coupled to a scanning electron microscope (JSM-6610LV, JEOL, Tokyo, Japan). Forty samples were examined, with irradiation performed by a 70 μm diameter electron beam at 25 kV and automatic current adjustment, and spectra were collected for 100 s per point. For each sample, elemental mapping of carbon (C), oxygen (O), sodium (Na), magnesium (Mg), phosphorus (P), chlorine (Cl), and calcium (Ca) was carried out along a linear array of 40 × 1 points with a 10 μm step size. Spectral data were processed using OXFORD AZTEC EDS software, and for descriptive analyses, the weight concentrations of Ca, P, and O were specifically considered (Dos Santos et al. 2024; Rafiee et al. 2024).

#### Raman spectroscopy

Raman spectroscopy analyses were performed using a spectrometer (Ocean Optics, Dunedin, FL, USA) equipped with a diode laser (λ = 785 nm) and a spectral resolution of 11 cm⁻¹, with measurements conducted at a laser power of 400 mW, an acquisition time of 5 s per spectrum, and three repetitions per region, yielding an averaged spectrum for each analyzed area. Spectral processing was carried out using a Python-based routine, with spectra organized by experimental group and average spectra for each region calculated using Ocean Optics Spectra Suite software (Dunedin, FL, USA). The main vibrational bands analyzed included 438 cm⁻¹ (phosphate), 582 cm⁻¹ (vibrational modes ν₁, ν₃, and ν₄ of hydroxyapatite), 960 cm⁻¹ (vibrational stretching of phosphate ions), 1070 cm⁻¹ (carbonate), 1270 cm⁻¹ (vibrational modes of CO₂), and 1450 cm⁻¹ (amide III, CH₂, and amide I). In addition, the ratio between the carbonate and phosphate peaks (1070/960 cm⁻¹) was quantified considering both peak area and intensity.

### Statistical analysis

Descriptive analyses were performed for SEM and AFM data. Quantitative data were statistically analyzed using GraphPad Prism 5 software (GraphPad Software, San Diego, California, USA). Initially, data distribution was assessed through normality test (Shapiro-Wilk) and homogeneity of variances was verified using Levene’s test, adopting a significance level of α = 0.05. Subsequently, data were submitted to One-Way ANOVA, followed by Tukey’s post hoc test, also adopting α = 0.05 as the level of statistical significance.

## Results

### Relative microhardness and remineralization potential

The relative microhardness analysis (Table 1) revealed negative values across all groups, indicating incomplete remineralization. Group G10 (control without caries + RT) presented the highest relative microhardness, significantly different from the other groups (*p* < 0.05), while no statistical differences were found among the remaining groups. Pre-RT, G1 (biosilicate suspension) showed the highest microhardness, differing significantly from G3 (*p* < 0.001) and G5 (*p* < 0.001), but not from other groups (*p* > 0.05). G3 and G5 (gel-based) did not differ from each other; however, G5 (chitosan-based) differed from G7 (*p* = 0.0205) and G9 (*p* = 0.0248). Post-RT, G4 (biosilicate gel) exhibited the highest relative microhardness, significantly different from all other groups except G8 (fluoride), which showed values similar to the remaining treatments. G6 and G9 showed no significant differences between them or with G2. Regarding remineralization potential, groups G9 and G10 were excluded as controls. After caries simulation and RT, the lowest remineralization potential was observed in G1 and G2, with similar results between them and differing from all other groups, except G6 (*p* > 0.05).

**Table 1:** Relative microhardness and Remineralization potential of samples subjected to the caries simulation protocol, radiotherapy, and treated with biosilicate suspension and gel (G1-G4), chitosan-based gel (G5 and G6), or fluoridated gel (G7 and G8) pre- and post-RT, and controls (G9 and G10)

Groups	Mean (SD)		
	Relative Microhardness	Relative Microhardness Pre and Pos-RT	Remineralization Potential
*G1*	−98.70 (0.36) ^B^	−98.70 (0.36) ^C^	0.58 (0.35) ^B^
*G2*	−98.58 (0.43) ^B^	−98.58 (0.43) ^C^	0.63 (0.34) ^B^
*G3*	−98.07 (0.39) ^B^	−98.07 (0.39) ^AB^	1.16 (0.38) ^A^
*G4*	−97.85 (0.50) ^B^	−97.85 (0.50) ^A^	1.29 (0.50) ^A^
*G5*	−97.97 (0.44) ^B^	−97.97 (0.44) ^A^	1.21 (0.43) ^A^
*G6*	−98.26 (0.53) ^B^	−98.26 (0.53) ^BC^	1.01 (0.45) ^AB^
*G7*	−98.38 (0.45) ^B^	−98.38 (0.45) ^BC^	1.15 (0.66) ^A^
*G8*	−98.07 (0.43) ^B^	−98.07 (0.43) ^AB^	1.15 (0.36) ^A^
*G9*	−98.37 (0.25) ^B^	−98.37 (0.25) ^BC^	-
*G10*	−8.5 (18.5) ^A^	-	-

One-way ANOVA and Tukey’s test (*p* < 0.05). Different letters indicate statistically significant differences (*p* < 0.05). SD: Standard deviation. Reference values (mean remineralization potential): G9: 1.1 (0.8); G10: 116 (760).

### Surface roughness alteration

The comparison of surface roughness alteration is presented in Table 2. The raw roughness values showed a mean of 0.15 μm. Upon analyzing the groups after the caries simulation process and RT, the lowest surface roughness values were observed in G10 (*p* < 0.05). No statistically significant differences were found among the other groups.

**Table 2:** Surface Roughness of samples subjected to the caries simulation protocol, radiotherapy, and treated with biosilicate suspension and gel (G1-G4), chitosan-based gel (G5 and G6), or fluoridated gel (G7 and G8) pre- and post-RT, and controls (G9 and G10)

Groups	Mean (SD)
*G1*	2.68 (0.89) ^A^
*G2*	2.40 (0.67) ^A^
*G3*	2.49 (0.46) ^A^
*G4*	2.31 (0.67) ^A^
*G5*	2.37 (0.93) ^A^
*G6*	2.35 (0.91) ^A^
*G7*	2.46 (0.67) ^A^
*G8*	2.56 (0.90) ^A^
*G9*	2.61 (1.13) ^A^
*G10*	0.03 (0.04) ^B^

One-way ANOVA and Tukey’s test (*p* < 0.05). Different letters among the groups indicate statistically significant differences (*p* < 0.05). SD: Standard Deviation.

### SEM

**Fig. 1:**
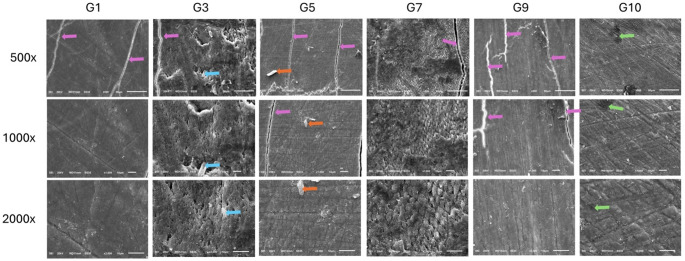
Scanning electron microscopy analysis at magnifications of 500x, 1000x, and 2000x of pre-RT (G1, G3, G5, and G7) and controls with caries + RT (G9) and without caries + RT (G10). Green arrows: microcavities; Pink arrows: cracks; Blue arrows: Biosilicate particles; Orange arrows: Chitosan particles

**Fig. 2:**
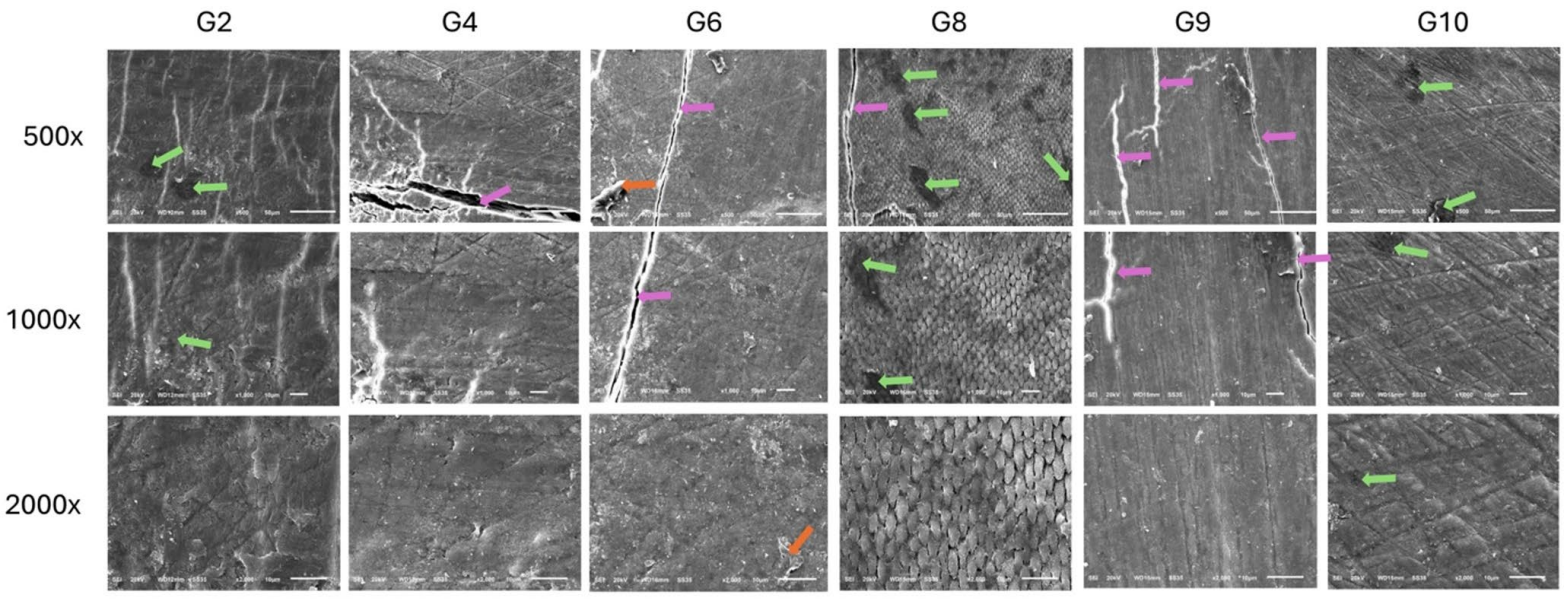
Scanning electron microscopy analysis at magnifications of 500x, 1000x, and 2000x of pos-RT (G2, G4, G6, G8) and controls with caries + RT (G9) and without caries + RT (G10). Green arrows: microcavities; Pink arrows: cracks; Blue arrows: Biosilicate particles; Orange arrows: Chitosan particles

SEM analysis qualitatively demonstrated that RT increased enamel porosities, cracks, and microcavities in G10, with G9 (ACP + RT) exhibiting more severe structural damage. Pre-RT **(**Fig. 1**)**, G1 and G5 showed surface porosities and cracks without microcavities; G5 additionally presented dense particle deposits. G3 exhibited surface degradation with particle accumulation, whereas G7 showed wide cracks and enamel disruption. Post-RT **(**Fig. 2**)**, more uniform surface coverage was observed in G2, G4, and G6, although G2 and G8 exhibited microcavities and G4 and G6 displayed extensive cracking, with particle deposits also present in G6. G8 showed a layered pattern resembling the pre-RT surface of G7 (“fish scale” appearance). Qualitative analysis indicated that G1, G3, and G5 provided superior pre-RT protective effects, while G2, G4, and G6 demonstrated greater post-RT remineralization potential. G7 and G8 exhibited limited protective and remineralizing capacity. Overall, particle deposition was associated with both protective and remineralizing effects on enamel surfaces.

### AFM

**Fig. 3:**
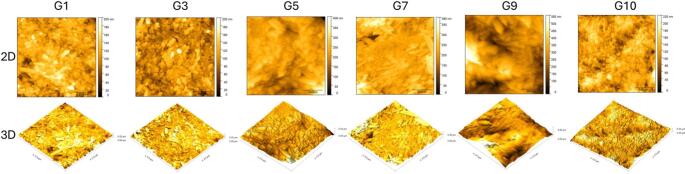
Two-dimensional (2D) and three-dimensional (3D) atomic force microscopy analyses of the pre-RT (G1, G3, G5, G7) and the controls with caries + RT (G9) and without caries + RT (G10)

**Fig. 4:**
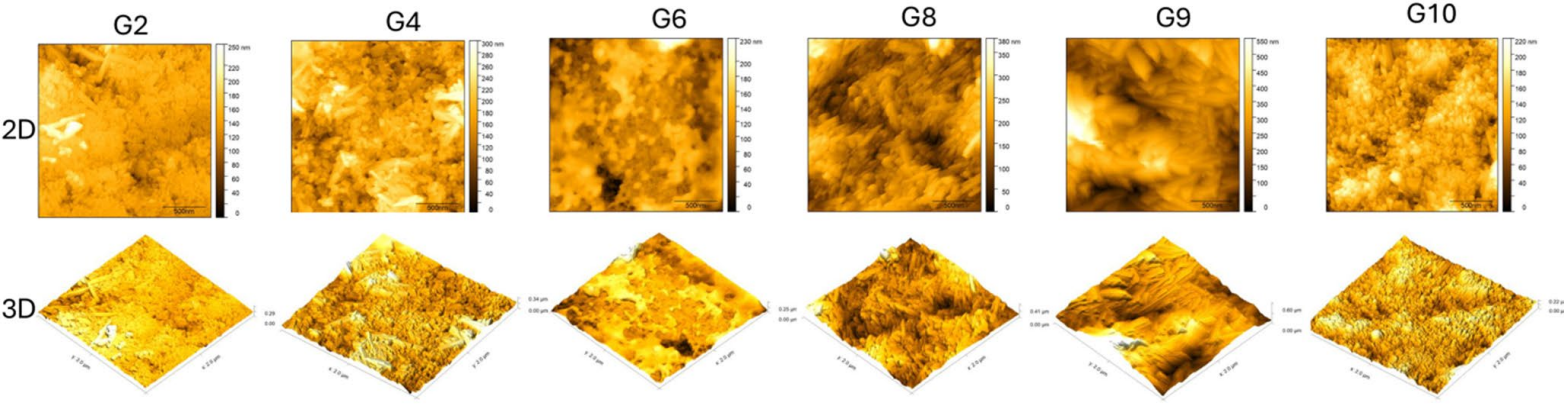
Two-dimensional (2D) and three-dimensional (3D) atomic force microscopy analyses post-RT (G2, G4, G6, and G8) and the controls with caries + RT (G9) and without caries + RT (G10)

AFM images qualitatively demonstrated greater surface irregularities in G9 (caries simulation + RT without treatment), whereas G10 (RT only) showed a porous, irregular surface without detectable material deposition. Pre-RT **(**Fig. 3**)**, G1 and G3 exhibited a uniform whitish–glassy particulate layer; G5 presented a more homogeneous but sparser layer; G7 displayed finer, more dispersed particles. Post-RT **(**Fig. 4**)**, G2 showed sparse yet regular coverage, G4 exhibited denser deposition with reduced damage depth relative to G10, and G6 demonstrated the least surface degradation with heterogeneous coverage interspersed by depressions; G8 displayed substantial structural loss comparable to G9. Overall, particle deposition in treated groups was associated with improved post-RT surface integrity relative to untreated controls.

### EDS

Table 3 presents the mean and standard deviation of the weight% of calcium, phosphorus, and oxygen. No statistically significant differences were detected among the groups for any of the evaluated elements.

**Table 3:** EDS analysis of the groups (G1 - G10) for the elements Calcium (Ca), Phosphorus (P), Oxygen (O), and the elemental ratios Ca/P, Ca/O, and O/P, showing mean values and standard deviation

Groups	Mean (SD)					
	Ca	*P*	O	Ca/*P*	Ca/O	O/*P*
*G1* ^*A*^	35.20 (1.71)	15.80 (0.42)	46.50 (0.28)	2.23 (0.06)	0.76 (0.04)	2.94 (0.09)
*G2* ^*A*^	33.40 (1.28)	15.30 (0.46)	47.00 (0.70)	2.18 (0.05)	0.71 (0.03)	3.07 (0.13)
*G3* ^*A*^	32.30 (0.64)	15.10 (0.32)	47.30 (1.64)	2.14 (0.01)	0.68 (0.02)	3.13 (0.09)
*G4* ^*A*^	33.30 (1.30)	15.50 (0.42)	45.80 (1.64)	2.15 (0.09)	0.73 (0.04)	2.95 (0.08)
*G5* ^*A*^	27.40 (4.00)	12.80 (1.91)	44.60 (1.60)	2.14 (0.04)	0.61 (0.07)	3.48 (0.42)
*G6* ^*A*^	27.70 (1.71)	13.00 (0.91)	44.90 (0.64)	2.13 (0.04)	0.62 (0.04)	3.45 (0.21)
*G7* ^*A*^	32.80 (0.87)	15.00 (0.39)	46.10 (1.16)	2.19 (0.07)	0.71 (0.03)	3.07 (0.09)
*G8* ^*A*^	33.20 (0.71)	15.50 (0.38)	46.70 (1.01)	2.14 (0.02)	0.71 (0.03)	3.01 (0.10)
*G9* ^*A*^	32.40 (1.04)	15.20 (0.57)	46.70 (1.20)	2.13 (0.03)	0.69 (0.03)	3.07 (0.17)
*G10* ^*A*^	33.50 (0.55)	15.70 (0.41)	44.30 (1.98)	2.13 (0.08)	0.76 (0.04)	2.82 (0.07)

Different letters indicate statistical differences

### Raman spectroscopy

**Fig. 5:**
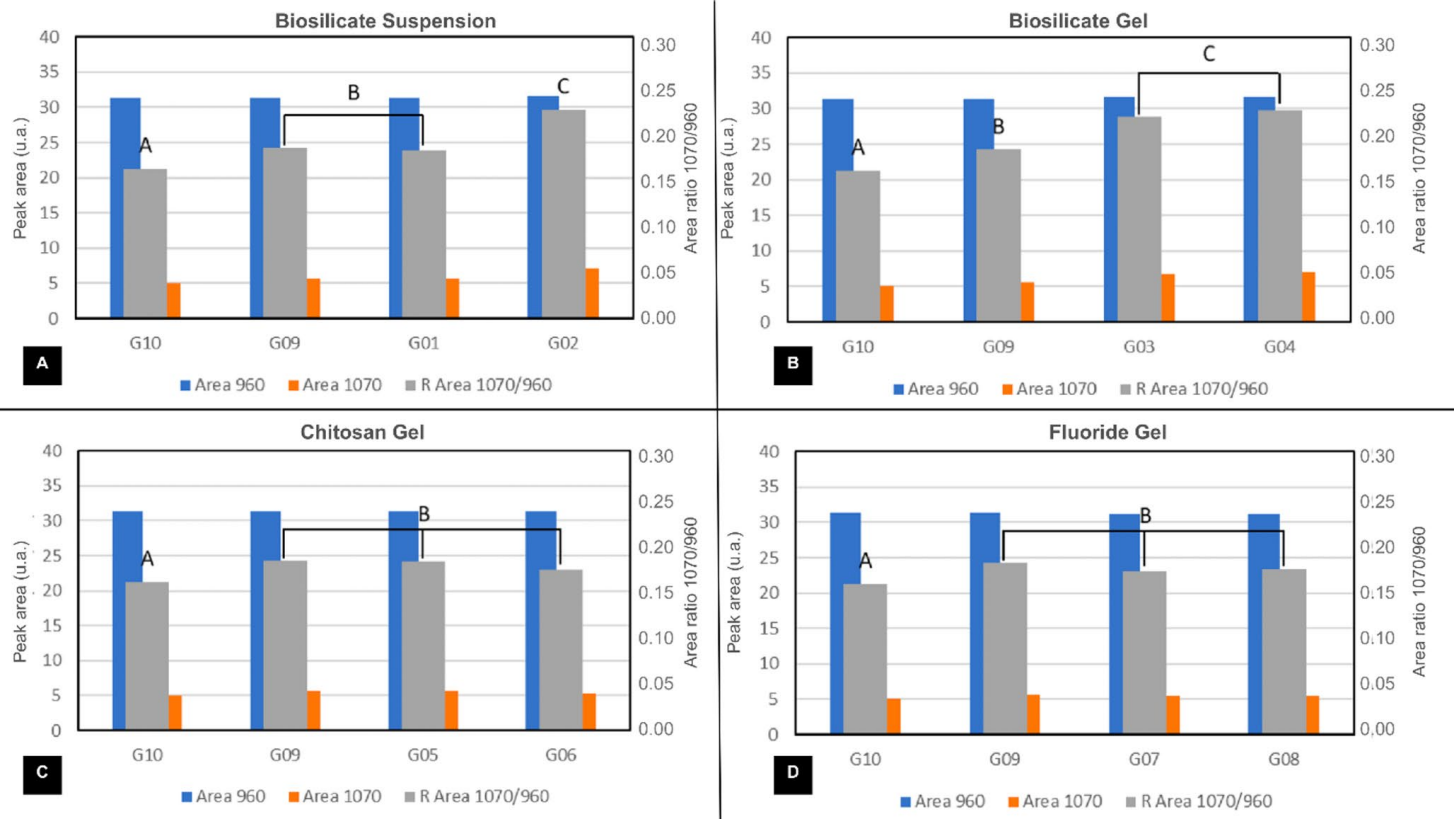
Peak areas 960 cm⁻¹ and 1070 cm⁻¹ for the control groups (G9 and G10) and treatments (G1-G8), for Biosilicate Suspension (**A**), Biosilicate Gel (**B**), Chitosan Gel (C) and Fluoride Gel (D). Analyses were conducted using One-Way ANOVA and Tukey’s test (*p* < 0.05). Different letters among the groups indicate statistically significant differences (*p* < 0.05)

Spectral changes reflected alterations in the structural and chemical composition of dental tissues, requiring analysis of peak intensities and total spectral area. The vibrational modes of carbonate (1070 nm) and phosphate (960 nm) in control groups (G9 and G10) and treated groups (G1 to G8) are shown in Fig. 5. A significantly higher carbonate/phosphate ratio was observed in G9 compared to G10, indicating greater demineralization. G1 and G2 also exhibited elevated ratios **(**Fig. 5-A**)**; however, G1 presented significantly lower values than G2, G3, and G4, suggesting reduced demineralization. No significant differences were observed among G2, G3, and G4 **(**Fig. 5-B**)**. G5 and G6 showed no significant changes in spectral intensity, but a reduction in the carbonate/phosphate ratio was observed, suggesting potential protective effects against demineralization (Fig. 5-C**)**. G7 and G8 demonstrated reduced carbonate/phosphate ratios **(**Fig. 5-D**)**, statistically similar to G9 but significantly different from G10, emphasizing the persistent damage caused by RT and caries simulation. None of the treatments exhibited outcomes superior to G9, with comparable statistical results.

## Discussion

The present study evaluated the protective and remineralizing effects of biosilicate, applied as gel and suspension, chitosan gel, and fluoride gel on enamel exposed to RT and ACP. Clear alterations in the topographic, mechanical, and chemical properties of enamel were observed after RT/ACP exposure, including reductions in microhardness and morphological changes identified through SEM, AFM, and Raman spectroscopy. These findings are consistent with mechanisms previously described for radiation-induced dental damage, in which both direct alterations to the crystalline structure of enamel and indirect effects mediated by free radicals generated during water radiolysis contribute to the disruption of hydroxyapatite organization and increase enamel susceptibility to demineralization (Gonçalves et al. 2014; Morgan et al. 2018; Kudkuli et al. 2020; Yao et al. 2024). In general, biosilicate and chitosan showed greater capacity to reduce mineral loss and partially preserve enamel microhardness both before and after irradiation, while fluoride presented better performance when applied before RT but limited capacity to repair damage once irradiation had occurred. These observations highlight the importance of preventive strategies capable of reducing radiation-induced enamel damage in patients undergoing head and neck radiotherapy. Early preventive care may help delay demineralization and contribute to maintaining oral function and quality of life (Yuwanati et al. 2021; De Cicco et al. 2021).

Biosilicate has been frequently described as a promising biomaterial for dental remineralization (Panzeri Pires-de-Souza et al. 2015; Chinelatti et al. 2017; De Cicco et al. 2021; Ferreira et al. 2022; Abreu et al. 2023; Geng et al. 2024). This bioactive glass-ceramic initiates a sequence of physicochemical reactions when it comes into contact with oral fluids, resulting in the formation of a carbonated hydroxyapatite layer that resembles the mineral structure of natural tooth tissue (Panzeri Pires-de-Souza et al. 2015; De Cicco et al. 2021). Its mechanism involves rapid ionic exchange at the material surface, followed by silica-rich layer formation, release of calcium and phosphate ions, and a local increase in pH, promoting hydroxycarbonate apatite precipitation on enamel under conditions favorable for mineral deposition (Abreu et al. 2023; Araujo et al. 2023).

Biosilicate-based materials may also present advantages over conventional remineralizing therapies because they act as bioactive ion-releasing systems capable of promoting ionic supersaturation at the enamel surface. The release of calcium, phosphate, sodium, and silicate ions favors the precipitation of hydroxycarbonate apatite and contributes to the formation of a mineralized surface layer (Panzeri Pires-de-Souza et al. 2015; Chinelatti et al. 2017). Unlike fluoride therapies, which mainly enhance enamel resistance to acid dissolution, biosilicate may also serve as a direct source of mineral ions involved in remineralization dynamics (Yao et al. 2024). In addition, the continuous ion exchange promoted by bioactive glass particles may contribute to partial structural recovery and improvement of enamel mechanical behavior under demineralizing conditions (Abreu et al. 2023; Geng et al. 2024).

These mechanisms may be particularly relevant in irradiated enamel, where ionizing radiation can disturb the crystalline organization of hydroxyapatite and increase mineral solubility, creating conditions in which materials capable of releasing calcium and phosphate ions can facilitate remineralization (Gonçalves et al. 2014; Kudkuli et al. 2020; Yao et al. 2024; Tamahara et al. 2025). In the present study, specimens treated with biosilicate exhibited reduced mineral loss when compared with the control group, supporting its protective role against radiation- and acid-related demineralization.

The formulation also influenced the performance of biosilicate. The gel form showed greater surface retention, which likely favored prolonged interaction with enamel and stabilization of the mineral layer, whereas the suspension produced a faster initial response but may require more frequent applications to maintain its effect (Pires-de-Souza et al. 2022; Abreu et al. 2023). Microscopic analyses supported these observations. SEM images revealed the deposition of dense glassy particles on the enamel surface, while AFM demonstrated a relatively homogeneous topography characterized by compact apatite-like microlayers. This microstructural arrangement may explain the improved resistance to acid challenge and the partial preservation of microhardness values observed in biosilicate-treated samples. EDS analysis, however, did not reveal marked differences in elemental composition among groups, suggesting that the remineralizing effect may initially occur mainly at the enamel surface and that longer exposure periods could be necessary for deeper mineral incorporation (Panzeri Pires-de-Souza et al. 2015).

Chitosan has also gained attention as a biomaterial for dental applications, particularly under conditions that promote enamel demineralization such as those associated with radiotherapy (Nimbeni et al. 2021; Lopes et al. 2023; Fathy et al. 2024). Derived from chitin, chitosan presents favorable biological properties including biocompatibility, biodegradability, antimicrobial activity, and a cationic charge that enables electrostatic interaction with negatively charged dental surfaces, facilitating adhesion to enamel and dentin (Nimbeni et al. 2021; Dur et al. 2022; Fathy et al. 2024). In this study, specimens treated with chitosan demonstrated partial preservation of surface microhardness following cariogenic challenge and irradiation. This effect may be related to the ability of chitosan to act as a reservoir for calcium and phosphate ions, supporting partial reconstruction of the mineral matrix (Nimbeni et al. 2021; Dur et al. 2022). However, because the formulation evaluated in the present study consisted of a combination of chitosan, casein phosphopeptides, and nanohydroxyapatite, the individual contribution of each component could not be independently determined. Therefore, the observed protective effects should be interpreted as the result of the combined physicochemical behavior of the formulation rather than the isolated action of a specific compound. Measurements of surface roughness did not differ significantly among groups, which is consistent with previous reports indicating that chitosan tends to promote relatively uniform mineral deposition and surface stabilization rather than pronounced changes in surface morphology (Dalmolim et al. 2025).

Additional structural analyses reinforced the protective and surface-stabilizing potential of chitosan. SEM and AFM images showed relatively homogeneous surface deposits and a more regular topography when compared with untreated irradiated enamel. Raman spectroscopy identified phosphate-related peaks compatible with amorphous calcium phosphate or early hydroxyapatite formation, suggesting the initiation of mineral recovery processes. These findings agree with previous studies reporting that chitosan-based formulations, particularly when associated with calcium or phosphate derivatives, can promote biomimetic mineralization and stabilize early enamel lesions (Nimbeni et al. 2021; Lopes et al. 2023; Soliman et al. 2026); Stafin et al. 2026). EDS analysis showed a modest increase in the Ca/P ratio in the chitosan groups compared with demineralized controls, although without statistical difference relative to fluoride. This observation suggests that the effect of chitosan may be related mainly to stabilization of the enamel surface and modification of the ionic environment rather than extensive subsurface mineral incorporation (Dur et al. 2022). Considering its biocompatibility, antimicrobial characteristics, and mucoadhesive behavior, chitosan may represent a useful adjunct in preventive strategies for oncology patients, especially those experiencing xerostomia or mucosal sensitivity during radiotherapy (McComb et al. 2002; Ferlay et al. 2015; Gupta et al. 2015).

Fluoride remains one of the most established agents for the prevention of dental caries and has long been recommended for patients undergoing head and neck radiotherapy (Conti et al. 2022; Suwannapong et al. 2025). In the present study, fluoride treatment promoted partial preservation of enamel microhardness, indicating superficial remineralization and increased resistance to acid dissolution. This effect is mainly attributed to the formation of fluorapatite, a mineral phase that presents greater resistance to acidic conditions than hydroxyapatite (Wu et al. 2020; Yao et al. 2024). However, roughness analysis did not demonstrate significant improvement compared with untreated irradiated enamel, suggesting that fluoride alone may have a limited capacity to reverse structural damage caused by radiation. SEM and AFM images revealed irregular enamel morphology with fissures and so-called “fish-scale” defects, particularly after irradiation, indicating greater surface deterioration when compared with biosilicate- and chitosan-treated specimens (Suwannapong et al. 2025; Guzmán et al. 2025). Raman spectroscopy showed an increase in phosphate band intensity around 960 cm⁻¹, consistent with fluorapatite formation, although these changes were less pronounced than those observed in the biosilicate group (Guzmán et al. 2025). EDS analysis also showed no substantial increase in calcium or phosphorus levels, reinforcing the interpretation that fluoride acts mainly at the enamel surface (Yao et al. 2024). Therefore, the mechanical improvements observed in the present study should be interpreted as evidence of partial protective and surface-preserving effects rather than complete bulk remineralization. The absence of significant chemical differences in EDS analysis suggests that compositional alterations may have remained restricted to superficial enamel regions or below the detection threshold of the applied analytical methods within the evaluated experimental period.

Although none of the treatments consistently demonstrated outcomes superior to G9 across all evaluated parameters, biosilicate and chitosan generally showed more favorable trends regarding partial preservation of enamel structure and microhardness. Fluoride, although still valuable in providing superficial protection, demonstrated lower capacity to promote deeper mineral stabilization under the conditions simulated in this study. Although combination therapies were not evaluated in the present study, future investigations may explore whether biomaterials with complementary physicochemical mechanisms could provide complementary protective effects on irradiated enamel (Quero et al. 2025).

Current evidence supports the implementation of preventive oral care strategies before radiotherapy in order to reduce radiation-related dental damage and maintain enamel integrity during cancer treatment (Thariat et al. 2012; Gupta et al. 2015; Hong et al. 2018). In agreement with this concept, the present findings suggest that remineralizing agents may be particularly beneficial when applied before radiotherapy, since preventive surface protection may reduce subsequent radiation- and acid-related enamel degradation. Although post-radiotherapy application demonstrated comparatively lower remineralization potential overall, biosilicate- and chitosan-based formulations still exhibited relevant protective effects, particularly when compared with fluoride, which remains one of the most commonly used preventive agents in oncology dental care. Continued application during radiotherapy and follow-up may also contribute to maintaining enamel protection and supporting remineralization; however, the ideal frequency, number of applications, and treatment duration should be established in future clinical studies.

From a broader perspective, strategies that reduce radiation-related oral complications may also contribute to global health efforts aimed at addressing the burden of noncommunicable diseases. The SGDs highlight the importance of reducing premature mortality and improving long-term well-being in patients affected by conditions such as cancer (Morgan et al. 2018; Niessen et al. 2018). Oral complications associated with head and neck radiotherapy may compromise nutrition, speech, and overall quality of life, reinforcing the importance of preventive interventions directed at dental tissues during cancer treatment. Oral health has increasingly been recognized as an important component of the broader global health agenda because of its close relationship with systemic health, health inequalities, and access to care (Sogi 2025). In this context, the development of bioactive materials capable of protecting dental tissues during radiotherapy may contribute not only to improved oral outcomes but also to broader strategies aimed at reducing treatment-related morbidity in cancer patients.

Some limitations should be considered when interpreting the findings of this study. Although bovine enamel was used in this study, this substrate is widely accepted in dental research due to its availability, larger surface area, and greater standardization, which contribute to reduced variability in experimental conditions. Comparative studies have demonstrated that, despite differences related to dietary adaptation—such as variations in prism organization, interprismatic structure, and Hunter–Schreger band patterns—bovine and human enamel share fundamental microstructural characteristics, including similar prismatic architecture and decussation patterns, supporting its use as a suitable experimental model (Wang et al. 2021). Thus, the use of bovine enamel does not represent a critical limitation but rather a methodological choice that enables controlled in vitro investigation. However, caution is warranted when extrapolating these findings to clinical conditions, given the known differences in mechanical behavior and structural organization between substrates.

Although the irradiation protocol was designed to simulate the daily fractionation commonly used in clinical head and neck radiotherapy, the in vitro model cannot fully reproduce the complexity of the oral environment. Factors such as salivary flow, oral microbiota, dietary habits, and mechanical wear may influence both the progression of radiation-related dental damage and the effectiveness of remineralizing agents. In addition, the relatively short application period may underestimate the long-term remineralization potential of certain biomaterials, particularly biosilicate, which requires time for silica gel maturation and apatite crystallization. In addition, SEM and AFM analyses were interpreted qualitatively (Dos Santos et al., 2024), and quantitative morphometric parameters such as crack density, porosity distribution, or surface defect measurements were not evaluated. Therefore, the microscopic findings should be interpreted as complementary structural observations supporting the mechanical and spectroscopic analyses. Similarly, Raman spectroscopy analysis was limited to evaluation of relative phosphate- and carbonate-related band changes, while additional parameters associated with crystallinity and peak broadening were not investigated.

Future studies may explore optimized formulations, longer treatment protocols, and combinations of remineralizing agents with complementary mechanisms of action. For example, biosilicate associated with fluoride may combine bioactive ion release and apatite formation with increased enamel resistance to acid dissolution promoted by fluorapatite formation. Similarly, chitosan associated with calcium-phosphate complexes may enhance biomimetic mineral deposition and stabilization of early enamel lesions through ion retention and surface adhesion mechanisms. These combinations represent promising strategies for improving remineralization effectiveness in irradiated enamel.

The incorporation of remineralizing strategies into oncology dental care requires collaboration among dentists, oncologists, and other healthcare professionals. Preventive protocols should consider the stage of cancer therapy, the level of dental compromise, and patient adherence to oral hygiene recommendations. By preserving dental tissues and maintaining oral health during radiotherapy, these approaches may contribute to better quality of life and improved treatment outcomes for patients with head and neck cancer.

## Conclusion

The results indicate that the combination of artificial caries challenge and radiotherapy promotes significant structural and mechanical alterations in dental enamel, including reduced microhardness and surface changes associated with mineral destabilization. Biosilicate and chitosan-based formulations demonstrated more favorable trends regarding partial preservation of enamel microhardness and surface integrity under the evaluated experimental conditions, whereas fluoride showed mainly superficial protective effects after irradiation. Microscopic and spectroscopic analyses demonstrated surface modifications suggestive of partial mineral stabilization in treated groups, suggesting that bioactive materials may represent promising strategies to protect dental tissues and mitigate radiation-related enamel damage during head and neck radiotherapy.

## Data Availability

The data that support the findings of this study are available from the corresponding author upon reasonable request.
